# Changing trends in carrier screening for genetic disease in the United States

**DOI:** 10.1002/pd.4647

**Published:** 2015-07-27

**Authors:** Shivani B. Nazareth, Gabriel A. Lazarin, James D. Goldberg

**Affiliations:** ^1^ CounsylSouth San FranciscoCAUSA

## Abstract

Genetic disease is the leading cause of infant death in the United States, accounting for approximately 20% of annual infant mortality. Advances in genomic medicine and technological platforms have made possible low cost, pan‐ethnic expanded genetic screening that enables obstetric care providers to offer screening for over 100 recessive genetic diseases. However, the rapid integration of genomic medicine into routine obstetric practice has raised some concerns about the practical implementation of such testing. These changing trends in carrier screening, along with concerns and potential solutions, will be addressed. © 2015 The Authors. *Prenatal Diagnosis* published by John Wiley & Sons Ltd.

## Abstract 2

**What's already known about this topic?**

- Expanded carrier screening is being widely used to screen for a large number of genetic disorders.

**What does this study add?**

- This study reviews the changing technology being utilized for expanded carrier screening and presents an argument for pre‐conceptual screening.

‘It is likely that within a few decades people will look back on our current circumstance with a sense of disbelief that we screened for so few conditions…they will also be puzzled and dismayed, as I am now, that our healthcare system put so many couples in an unnecessarily difficult position, by not identifying their carrier status until a pregnancy was already underway’^1^ ‐*Francis Collins, M.D., Ph.D., Director National Institutes of Health*

## Introduction

Screening for genetic disease has been a long established part of preconception and prenatal care. One model for carrier screening has been the community wide screening programs for Tay–Sachs disease (TSD) established in the 1970s.^2^ These programs focused on a single severe disease that had an increased carrier frequency in a recognized ethnic group, Eastern European Ashkenazi Jews. Wide implementation of these programs has significantly reduced the incidence of TSD in this ethnic group, with the majority of affected infants currently being from other ethnic groups.

Another disorder that has been widely screened for is cystic fibrosis (CF). It has been over a decade since the American College of Obstetricians and Gynecologists (ACOG) and the American College of Medical Genetics and Genomics (ACMG) initiated guidelines for prenatal and preconception carrier screening of cystic fibrosis. Initial guidelines recommended screening Caucasian individuals or those with a family history of cystic fibrosis. In April 2011, the ACOG Committee on Genetics updated their CF screening guidelines, stating that it has become increasingly difficult to classify individuals with CF into distinct ethnic categories.^3^ The Committee agreed that it is reasonable to offer CF to all couples planning a pregnancy because it allows them to consider all reproductive options, including pre‐implantation genetic diagnosis, prenatal diagnosis, gamete donation, or adoption.

Given the long history of carrier screening, there are still relatively few guidelines available to providers, resulting in inconsistent practices. In addition, there are conflicting recommendations among organizations (ACOG, ACMG) that have published guidelines. Both organizations recommend screening for cystic fibrosis, Ashkenazi Jewish disorders, and Tay–Sachs disease but ACMG list more disorders on its ‘Jewish’ panel.^3^, ^4^, ^5^, ^6^, ^7^ ACOG also recommends ethnicity‐based screening for hemoglobinopathies.^8^ Only ACMG recommends screening for spinal muscular atrophy.^9^ Neither organization recommends screening for fragile X syndrome, unless the family history suggests it or the patient specifically requests it.^10^, ^11^

Outside of the United States guidelines are more limited. Australia supports universal screening for cystic fibrosis carriers in pregnancy, while guidelines in Canada and United Kingdom do not support this approach.^12^, ^13^, ^14^ These guidelines are in part driven by cost considerations, and the current availability of cost‐effective gene panels suggests the need to revisit the way screening is offered to patients.

### Limitations of ethnicity‐based guidelines

Advances in medicine have continually prompted physicians to reevaluate well established but outmoded screening protocols in favor of new protocols. As an example, maternal age has been a cost‐effective means of screening and identifying ‘high‐risk’ women who could benefit from invasive prenatal testing by amniocentesis or chorionic villus sampling. Improvements in first and second trimester maternal serum screening options have allowed obstetricians to gradually shift away from age‐based screening. Today, all pregnant women are offered maternal serum screening, and any woman identified as high‐risk based on biochemical and ultrasound markers is offered prenatal diagnosis, irrespective of age.^15^ This trend will continue with the recent developments in cell‐free fetal DNA screening, making the term ‘advanced maternal age’ somewhat obsolete.

Similarly, ethnicity‐based screening for genetic disorders has been the most cost‐effective means of identifying couples at risk of bearing affected children. To date, the additive expense of single‐gene tests made population‐wide screening for recessive diseases cost‐prohibitive when those diseases largely occur in specific ethnic groups. Using ethnicity as a screen thus enabled carrier testing to capture individuals most likely to have a mutation.^5^ However, mixed ethnicity, adoption, and unknown ancestry compromise the application of the ethnicity‐based approach. The birth of babies with classically ‘Jewish’ disorders to non‐Jewish families exemplifies the pitfalls of defining genetic risks based on patients' self‐report and increasingly nebulous social constructs of race and ethnicity.^16^ The ACOG statement on CF carrier screening acknowledges the difficulty of assigning a single ethnicity to individuals as a justification for offering pan‐ethnic screening.^3^ Relying on ethnic categorization implies that most patients have some knowledge of their ancestral heritage, but data indicates that this is untrue.^17^

Additional moral concerns regarding ethnicity‐based screening have been raised by Ross.^18^ Her main concern is that newborn screening (NBS) is not ethnically driven, while most carrier screening continues to be so. She argues that to achieve equity with NBS, carrier screening should not be ethnically focused. She points out that provider‐determined or patient‐determined ethnicity is notoriously inaccurate and may vary depending on the reasons for ethnicity determination as seen in various antenatal screening studies. Ross argues that universal newborn screening is more equitable than ethnicity‐based screening citing many reasons, including the fact that universal screening avoids missed cases of rare disorders where early intervention reduces morbidity and mortality. In addressing the moral focus of newborn screening, the Secretary's Advisory Committee on Heritable Disorders in Newborns and Children (SACHDNC) acknowledges that genetic screening may be appropriate even without the availability of direct medical intervention, particularly if it will inform a family about future reproductive options.^19^ An ACMG Expert Panel referred to a ‘broadened concept of benefit’ that included the benefit of sparing families from the agony of the initial ‘diagnostic odyssey’ and the benefit of informing relatives of their increased genetic risks.^19^ Furthermore, they state that the public health benefit of reducing costs involved in the initial workup of a newborn with a rare disease of unknown etiology is an important consideration in the adoption of new technology. These benefits can be equally applied to universal preconception carrier screening.

## Expanded Carrier Screening

Recent developments in laboratory technologies have led to the commercial availability of expanded carrier screening (ECS) panels capable of assessing hundreds of mutations associated with genetic disease without an ethnic predilection or family history. Lazarin *et al.* reported on a targeted mutation panel which analyzed 417 disease causing mutations associated with 108 disorders.^20^ In their cohort of 23, 453 patients of mixed ethnicities, 24% were identified as carriers of at least one mutation. While many of the disorders on these panels are individually rare, the overall risk of having an affected offspring is 1 in 280 which is higher than the risk of having a child with a neural tube defect, for which screening is universal. Moreover, the cost of expanded carrier screening is often similar to or less than the cost of currently recommended ethnicity‐based panels.

The original methodology, and still commonly used in many ECS panels, is targeted genotyping. Targeted genotyping is limited by the limited number of mutations being tested for any specific disorder. Many of the disorders being tested for are rare, and there may be few known mutations in some ethnic groups. This may result in a minimal reduction in residual risk for the disorder in any tested individual. While the specific individual risk may be difficult to assign, this concept must be discussed with anyone being tested.

More recently, targeted sequencing by next generation sequencing (NGS) has been available. Bell *et al.* have reported a next generation carrier screening approach for 448 severe recessive disorders.^21^ While detecting an increased number of carriers compared to targeted genotyping, the authors point out the need for more extensive mutation databases to more accurately classify true disease causing mutations. This approach identifies an increased number of carriers and significantly increases sensitivity. Hallam *et al.* have also reported a NGS screening approach.^22^ Approximately 25% of the mutations they found would not have been detected with traditional targeted genotyping screening panels. In an assessment of NGS‐based screening for *HEXA* (Tay–Sachs disease) mutations in an ethnically diverse population, Hoffman, *et al.* found an 89–100% sensitivity when using NGS, compared with 68% for a fixed mutation panel.^23^

A successful expanded carrier screen, therefore, is determined by the breadth of diseases assayed and depth of the analysis. Balancing both factors is necessary; Srinivasan *et al.*, described the ‘long tail of Mendelian disease’ as exceeding in frequency the short list of common diseases, ultimately concluding that a universal screen ‘must assay a large number of different mutations, many of which are scarce outside of a particular subpopulation.’^24^ Application of NGS to expanded carrier screening appears to best hold the promise for an optimal test.

ECS enables at‐risk couple detection for an ever‐growing panel of diseases, and the identification of at‐risk couples is of course the ultimate goal of carrier screening. Assessing the severity of such diseases is a critical component of carrier screening panel construction. Practitioners will encounter unfamiliar diseases when performing ECS and may not immediately recognize a disease's severity; this can impair pre‐ or post‐test discussions with patients. Although severity has historically been subjectively defined, Lazarin, *et al.* developed a method for classifying a disease's severity into one of four ‘tiers’ by simple examination of that disease's clinical characteristics.^25^ This method may facilitate provider understanding and communication regarding ECS, as distinct diseases can now be related to one another—e.g., one can discuss carrier screening for Tay–Sachs disease, a Tier 4 ‘Profound’ disease, and others that have similar impact.

## Timing of Carrier Screening

Preconception genetic screening is not a new concept and has been a vital part of the Jewish community since the 1970s, and as a part of the Dor Yeshorim program since the 1980s.^26^ These programs have significantly reduced the incidence of Tay–Sachs disease in Jewish babies. Many well‐recognized Jewish organizations, such as the Jewish Genetic Disease Consortium (JGDC), JScreen.org, and the Center for Jewish Genetics recommend complete screening for all individuals of Jewish heritage in a timely manner. In fact, rabbi education programs encourage carrier screening of young Jewish couples during premarital counseling.^27^ Focusing on the cultural needs of the Jewish community, these organizations endorse screening for larger disease panels, as opposed to the currently recommended ACOG panel of four, or ACMG panel of 9. This is because while carrier status for each individual Jewish genetic condition is rare, a study conducted by Mount Sinai Laboratory found the overall carrier rate for at least one condition on their 16‐disease Ashkenazi Jewish panel is at least 30%.^28^ Similarly, as expanded screening panels become readily available to the general population, the incidence of each individual disease will be rare, but the collective population risk approaches and may even exceed 20%.

Given the reduced cost of screening for expanded disease panels, the preconception screening model in the Jewish community sets an excellent precedent for similar screening in the general population. Implementing this screening as a component of the well‐woman examination, rather than waiting until pregnancy is proximate or underway, improves the standard of care by helping many patients make autonomous decisions and consider modern reproductive technologies.

Preconception genetic screening is defined as carrier testing for genetic disorders prior to pregnancy onset, such as at the annual OB/GYN well‐visit exam. Between the ages of 19 and 39, national surveys indicate that 84% of women have seen a health care provider within the previous year and most women of reproductive age pursue preventative health services annually.^29^ The well‐woman visit presents an opportunity to review family history and discuss the available testing options without the stress of an ongoing pregnancy.^30^ The ACOG Committee on Genetics supports screening in the preconception period over prenatal period. Since 2005, the committee has released four opinions all recommending preconception genetic screening.^3^, ^6^, ^30^, ^31^ Despite the opportunity to educate women before they conceive, only 1 in 6 family physicians or OB/GYN providers gives preconception care. This implies the need to better understand the barriers to routine implementation of preconception health and education. A study by Jack *et al.* reviews these barriers in detail which include lack of clinical training on health promotion. The study suggests a technological solution to educate patients virtually and potentially alleviate common concerns around lack of preconception care.^32^

## Genetic Counseling and Informed Consent

Informed decision‐making with respect to carrier screening is important, but face‐to‐face counseling with a genetic counselor is impractical in the context of population screening—there simply are not enough genetic professionals.^33^ Earlier this year, a Joint Statement of the American College of Medical Genetics and Genomics, American College of Obstetricians and Gynecologists, National Society of Genetic Counselors, Perinatal Quality Foundation, and Society for Maternal–Fetal Medicine was issued regarding expanded carrier screening.^34^ The concept was defined as ‘all individuals, regardless of race or ethnicity, are offered screening for the same set of conditions.’ In this statement, the authors explicitly state that traditional pre‐test counseling with a full explanation of the characteristics of each disease on the panel is neither ‘practical or necessary.’

This reality forces us to consider creative, cost‐efficient solutions to providing informed consent without sacrificing quality. The notion that genetic testing requires more patient hand‐holding than traditional laboratory testing, particularly for recessive conditions, is an outdated and paternalistic approach to a rapidly growing field. James Evans instead suggests that the shortage of clinical genetics professionals may actually benefit the field by forcing genetics to become an integral component of general medicine.^35^ Nurses, physician assistants, and other medical professionals will be required to help navigate the screening process and service patient needs.

Genetic counselors will undoubtedly remain crucial to the provision of complex counseling and test interpretation, but pre‐test counseling for recessive diseases is far from complex. Francis Collins spoke at the 2011 ACOG Annual Clinical Meeting about the inevitable integration of carrier screening into routine obstetric practice. A survey of 104 obstetricians across the nation with respect to CF screening suggests that 87.7% were familiar with screening guidelines, and 82.3% could interpret basic test results.^36^ Although educational efforts to keep obstetricians up‐to‐date with genetic screening will be necessary, it is unreasonable to assert that traditional genetic counseling should be required for all such testing. Instead, genetic counselors can serve as a resource for more complicated findings that go beyond a physician's scope of understanding. Continuing medical education can also address gaps in knowledge that are inevitable in such a rapidly growing field of medicine.

## Anxiety and Stigmatization

If we are to move toward a more integrated approach to genetics within the field of medicine, any issues of anxiety and stigmatization surrounding genetic information must be removed as a barrier to adoption. Concerns about raised anxiety in carriers of recessive diseases seem to be overestimated. Lewis, *et al.* conducted a systematic review of 20 studies of the psychological impact of carrier testing for autosomal recessive and x‐linked genetic disorders.^37^ Their study identified several factors that influence emotional reactions to carrier testing: mode of inheritance, existing coping mechanisms, gender, personal connection to a particular disease, and stage of life. Longitudinal studies in this systematic review found no significant difference in anxiety between carriers and non‐carriers of cystic fibrosis.

Not surprisingly, patients who already had an affected child experienced feelings of guilt and shame associated with their positive carrier status and the birth of the affected child. In contrast, anxiety in those who underwent preconception carrier testing as part of routine screening largely dissipated within six months. The findings are similar with respect to feelings of stigmatization and guilt associated with cystic fibrosis screening. No significant difference was noted between carriers and non‐carriers in the general population. These participants did not have any personal experience or family history of cystic fibrosis. In contrast, those who already had an affected child were more likely to experience guilt and self‐stigmatization.

It is worth noting that study participants were not pregnant at the time of screening and therefore did not feel pressure to make immediate prenatal testing decisions. Additional studies cite similar results to support the findings that anxiety, guilt, and stigma do not carry the weight presupposed by some medical professionals.^38^, ^39^, ^40^, ^41^ It is plausible that physicians and genetic counselors are actually projecting their own anxiety about the time investment and energy required to convey more information onto their patients. One potential solution is to lean on the availability of post‐test telephone genetic counseling provided by laboratories seeking to help patients better understand their results and next steps. This non‐traditional model of genetic counseling reduces the burden on physicians to take on added responsibilities, and it frees up counselors in busy clinical genetics settings to focus their expertise on more complex patient cases. Some laboratories routinely provide post‐test genetic counseling to all patients, allowing physicians to offer an optimal carrier screening experience.

## Summary

As the cost of genetic testing continues to decrease, justifying the omission of rare diseases from carrier screening panels becomes increasingly difficult. The availability of screening for hundreds of diseases for the cost of only one or two single‐gene carrier tests may even represent a moral imperative to at least *offer* expanded screening in a timely manner. Rare Mendelian genetic diseases are collectively present in 1 in 280 births, which is more common than Down syndrome.^24^ Unfortunately, evidence suggests that where guidelines are not specified, providers admit to only offering ‘extra’ testing when specifically requested by the patient. For example, Benn *et al.* in a 2012 survey of ACOG members reported that 15% regularly offer ECS and 52% provide it when requested by the patient.^42^ This places the onus of preventative health on the patient, rather than the provider. It is not the duty of the patient to keep abreast of advancements in genetic medicine. Author Emily Rapp, an Irish woman who lost her first son to Tay–Sachs disease, writes poignantly about the issue of parental access to information:

‘What I hope for other women is that they have the power to make their own decisions with as much information as it is possible to have, with respect to the specificity and complexity of their own circumstances, according to their own minds and hearts and not the dictates of another person's worldview’^42^

Educational efforts to promote awareness of genetic tests and dispel any concept of stigmatization associated with carrier status will serve the public well, particularly because technological advancements will only prove the old genetics adage that we are all carriers of something. Understandably, low detection rates for rare disorders are not ideal, as a negative result could provide false reassurance. The implementation of NGS testing will help improve the detection rate for many disorders. The alternative is not to offer the screening test at all, and this is an even more severe form of false reassurance. Instead, patient education should address the limitations of screening as risk‐reducing rather than risk‐eliminating. In the end, some degree of reassurance is better than the current acceptable practice of blind reassurance.

Even when couples express that they would not change the course of their pregnancy based on carrier screening, knowledge of positive carrier status imparts additional benefits. These include management of a high‐risk pregnancy, preparation for possible birth complications, and early intervention in the newborn period. The joint ACOG/ACMGG/NSGC/SMFM/PQF statement on expanded carrier screening is an important step toward recognition of carrier screening as a critical component of preconception and prenatal care. We conclude that the annual well‐woman visit is an ideal time to incorporate this practice. Without the immediate concern of an ongoing pregnancy, women and their partners can consider all of their reproductive options and decide how much genetic information they would like to have before starting a family. At‐risk couples can consider alternative approaches to starting a family and minimize their risks in accordance with their religious and moral convictions. Waiting until a pregnancy is already underway deprives patients of the time required to process genetic information, make autonomous decisions, and take advantage of modern reproductive technologies.

**What's Already Known About this Topic?**

- Expanded carrier screening is being widely used to screen for a large number of genetic disorders.

**What Does this Study Add?**

- This study reviews the changing technology being utilized for expanded carrier screening and presents an argument for pre‐conceptual screening.
